# Revolutionizing adult oral care: Silver diamine fluoride's minimally invasive role

**DOI:** 10.6026/973206300214724

**Published:** 2025-12-15

**Authors:** Dayana Gutierrez, Arias Marjorie, Pedraza Kelly, Garcia Ana C, Agostini Cristina, Konakalla Mahita

**Affiliations:** 1 Department of Dentistry, Doctor of Dental Surgery, University of Magdalena, Santa Marta, Colombia, South America; 2 Dental Medicine Department, Doctor of Dental Medicine, Universidad Nacional Pedro Henríquez Ureña (UNPHU), Santo Domingo, Dominican Republic; 3 Department of Dentistry, Doctor of Dental Surgery, Specialist in Periodontics, Universidad Antonio Nariño, Bogotá, Colombia, South America; 4 Department of Dentistry, Doctor of Dental Surgery, Universidad Santa Maria, Caracas, Venezuela, South America; 5 Department of Biology, College of Arts and Science, University of South Florida, Florida, USA; 6 Department of Dentistry, Institute of Dental Science, Academy Drs. Sudha and Nageswara Rao Siddhartha, Bachelor of Dentistry andhra Pradesh, India

**Keywords:** Silver diamine fluoride, dental caries, antimicrobial, adult oral health, minimally invasive dentistry, medically compromised adults, special needs dentistry, vulnerable populations, public health dentistry, preventive dentistry, palliative care

## Abstract

Dental caries remains one of the most common chronic diseases in adults worldwide, particularly among the elderly and medically compromised. Conventional restorative treatments are often inaccessible or contraindicated in these populations due to physical, cognitive, or financial limitations. Silver diamine fluoride (SDF) has emerged as a powerful tool as the dental field embraces minimally invasive options.

## Background:

Silver diamine fluoride (SDF) is a topical alkaline solution containing fluoride and silver ions that effectively prevents and arrests dental caries. Its dual actions, remineralize and antibacterial properties, work together to halt the progression of caries. SDF is especially relevant for patients with special needs, the elderly or young kids who may be uncooperative, due to its simplicity and noninvasive approach. It is also very common in low-income populations because of its low cost and high effectiveness. While initially approved by the Food and Drug Administration (FDA) as a fluoride treatment to manage hypersensitive teeth, SDF is now predominantly used for caries control. In the United States, the most commonly used SDF product is Advantage Arrest, composed of 38% silver diamine fluoride. SDF consists of three main components: Ag (silver), F- (fluoride) and NH_3_ (ammonia). The ammonia ions bind to silver ions, forming diamine-silver, a complex ion that is more stable than silver fluoride alone. This complex helps maintain a concentration of 38% for a prolonged period. Each component plays a crucial role in caries arrest. Silver provides antimicrobial action, inhibiting biofilm formation [1]. Fluoride prevents caries, promotes remineralization and inhibits demineralization. Lastly, the ammonia stabilizes the solution and forms complex ions. Silver ions in SDF disrupt bacterial cell membranes and denature intracellular proteins, leading to bacterial death. The antimicrobial activity forms a protective barrier, reducing susceptibility to microbial invasion and acids causing decay. Streptococcus mutans, a primary caries-causing bacterium, produces acids that demineralize tooth structure and promote biofilm formation. SDF has antimicrobial resistance to the cariogenic biofilm S. mutans. Not only does the SDF kill the bacteria, but it stops recolonization for at least a week following application, showing long-term reduction of bacterial activity on treated lesions, as demonstrated by Chu *et al.* Fluoride ions in SDF react with the calcium phosphate in tooth enamel, converting hydroxyapatite into fluorohydroxyapatite, more resistant to acid demineralization [2]. SDF application results in a hardened outer layer of dentin, which forms a sclerotic layer of silver-protein. This process increases resistance to further acid dissolution. Mei *et al.* demonstrated that SDF can arrest carious lesions without excavation. The outer layer of the tooth and the soft decayed dentin were strengthened. The elimination of S. mutans halted the progression of the carious lesion and was successfully arrested [3]. Therefore, it is of interest to examine the efficacy, accessibility and emerging implications of SDF in adult oral healthcare, particularly among vulnerable populations.

## Clinical indications and applications of SDF in adult patients:

Over the past decade, silver diamine fluoride has experienced significant growth due to its minimal cost and numerous benefits, particularly in adult dentistry. SDF is especially beneficial for patients who present with functional impairment and chronic diseases; it is also preferred for those at high risk and those facing economic, social, or functional barriers to accessing dental care. Cavity excavation is not necessary before SDF application, but clinicians must ensure that the surface is clear of debris and plaque. The area must be isolated with cotton rolls to maintain dryness during the procedure. SDF is applied topically and is widely recognized for its effectiveness in arresting and preventing carious lesions [4]. However, due to its characteristic black staining, it is highly important to obtain the patient's informed consent before proceeding with the application. The procedure involves placing one drop of 38% SDF in a dappen dish, after isolating the affected teeth and drying the tooth surfaces. SDF is then applied using a cotton-tipped applicator or microbrush for one to three minutes. After application, the SDF should completely dry before removing isolation. SDF will become milky white when in contact with saliva. A bitter or metallic aftertaste is typical. Prescribing high-fluoride toothpaste improved the outcomes of SDF treatment. The frequency of SDF application should be determined based on each patient's caries risk and clinical judgement [5]. Follow-up treatments within 24 months are essential to prevent the lesion from reactivation [6]. For patients capable of self-care with minimal comorbidities, it is recommended that subsequent treatment be administered at 3, 4, or 6-month intervals or on an annual basis of SDF. Frail older adults with chronic medical conditions or functional limitations may require more frequent reapplication. The maximum recommended safe dose is one drop per 10 Kg of body weight per visit [7]. Unlike traditional restorative treatment, SDF is quick, cost-effective, well-tolerated and does not require anesthesia or drilling, which reduces fear and anxiety in high-risk patients. It is accessible to dental professionals and can be done with proper training [8]. However, it has limitations, including a persistent black staining of the carious lesion, inability to restore lost tooth structure and the possibility for the lesion to revert within 24 months if not reapplied [6]. Clinical decisions regarding SDF use should account for each patient's risk factors and health status. For example, patients undergoing chemotherapy, those with severe cardiovascular disease, or those with limited mobility may not tolerate conventional restorative care. In such cases, SDF offers a stable and effective alternative. Its application is especially valuable when managing non-restorable teeth, high caries recurrence, or during hospice care to control infection and discomfort.

## Clinical criteria for choosing SDF over restorative treatment:

## Tooth/lesion characteristics:

[1] Nonrestorable lesions:

1) Extensive structural loss where restoration would fail to retain (e.g., severe root caries, deep cervical lesions).

2) Advanced decay in primary teeth near exfoliation.

3) Teeth with poor prognosis where definitive treatment (e.g., extraction) is delayed.

[2] Lesions accessible for topical application:

1) Class V, root caries, or open occlusal lesions.

2) Cavitated lesions those are asymptomatic or minimally symptomatic.

[3] Lesions without pulpal involvement:

1) No spontaneous pain, swelling, or signs of irreversible pulpitis or necrosis.

2) Radiographs show intact lamina dura and no periapical pathology.

## Patient-specific factors:

[1] Comorbidities or medical conditions contraindicating restorative care:

1) Cardiovascular instability, bleeding disorders, immune compromise.

2) Neurodevelopmental or cognitive impairments that limit cooperation.

3) Conditions requiring hospitalization or general anesthesia for dental care (e.g., severe autism spectrum disorder, uncontrolled epilepsy).

4) Terminal illness or palliative care situations.

[2] Dental anxiety or behavioral limitations:

1) Inability to tolerate local anesthesia, rotary instrumentation, or isolation.

2) Extremely uncooperative pediatric or geriatric patients (especially in community or nursing home settings).

[3] Socioeconomic or access barriers:

1) Limited access to dental clinics.

2) Financial hardship precluding restorative care.

3) Refugee, migrant, or underserved populations.

## Clinical indications to prioritize reapplication of SDF:

[1] High caries risk:

1) Active disease progression in other sites.

2) Frequent carbohydrate intake, poor oral hygiene, dry mouth.

[2] Lesions previously arrested but at risk of reactivation:

1) Black-stained caries that appear softer or show slight discoloration changes.

2) Marginal breakdown around previously treated areas.

[3] Recall appointments show new or progressing lesions:

a. Indicates ongoing high-risk environment.

[4] Recommended reapplication intervals:

1) Every 6 months for high caries risk individuals.

2) Every 3 months if lesion is active or early SDF application.

3) Annually in stable patients with arrested lesions [9, 10, 11, 12, 13].

## Effectiveness of SDF in arresting root caries and cervical lesions in older adults:

Since the 1950s, the rate of edentulism has declined and many older adults now retain some or all of their natural teeth [14]. However, this trend is accompanied by an increased risk of dental caries, particularly root caries, due to age-related salivary reduction, dietary changes and root surface exposure from gingival recession. Root caries is an emerging global health concern amongst the elderly. Restorations in this population often fail due to recurrent decay at the gingival margins. As aging brings a combination of medical, functional and dexterity and mobility changes in patients as they age, along with poor nutrition, root caries increase exponentially. One of the most significant advantages of SDF is its ability to prevent and arrest root caries lesions as it eliminates cariogenic bacteria, primarily Streptococcus mutans and promotes remineralization of dentin. When left untreated, root caries can lead to tooth loss and pose life-threatening situations to elderly patients. Case reports have documented that dislodged prosthetic bridges caused by underlying root caries have been aspirated, resulting in severe consequences or even fatalities [15]. Emerging research suggests that oral hygiene instruction combined with annual applications of SDF solution may be more effective in preventing root caries than relying solely on traditional methods, such as meticulous toothbrushing and the use of fluoride toothpaste [16].

## Periodontal and endodontic considerations in the use of silver diamine fluoride (SDF):

Silver diamine fluoride (SDF) has been extensively studied for its effectiveness in arresting dental caries, but recent research suggests potential benefits in periodontal and endodontic applications. In the periodontal context, SDF has been shown to improve parameters such as gingival inflammation, bleeding and plaque accumulation. Although its mechanism of action is not fully understood, it is believed that its antimicrobial effect, particularly against Streptococcus mutans, Porphyromonas gingivalis and Aggregatibacter actinomycetemcomitans, helps reduce bacterial load, thereby decreasing gingival inflammation and bleeding. Additionally, SDF forms a protective layer on the tooth surface, making it more difficult for plaque and biofilm to adhere, major etiological factors of gingivitis and periodontitis. A clinical trial in older adults demonstrated that application of 38% SDF significantly reduced gingival inflammation and plaque accumulation compared to a control group. This effect may also be attributed to a reduction in dentin hypersensitivity, which enables patients to perform oral hygiene more effectively, thereby improving periodontal health [17]. In endodontics, SDF has shown the ability to occlude dentinal tubules, reducing sensitivity and serving as a barrier against bacterial invasion. Shimizu observed silver deposits within the tubules following SDF application, increasing resistance to caries recurrence [18]. Although findings have been variable, this sealing property has also been linked to antibacterial action against endodontic pathogens such as Enterococcus faecalis [19]. Several studies have investigated using a diluted 3.8% SDF solution as an intracanal irrigant. This concentration has been found to occlude tubular orifices after smear layer removal, demonstrating comparable efficacy to sodium hypochlorite (NaOCl) in some trials, with higher substantivity, similar to chlorhexidine. Nevertheless, results remain contradictory and further clinical trials are required to validate its routine use as an irrigant or interappointment medication [20]. While SDF may cause pulpal necrosis when applied directly to vital pulp tissue, studies have indicated that indirect applications are biocompatible, resulting in mild reversible inflammation and stimulating tertiary dentin formation [21]. SDF represents a promising adjunct in periodontics and endodontics due to its antimicrobial, desensitizing and dentinal tubule-sealing properties. However, its clinical use in endodontic therapy requires further evidence to establish safe and effective protocols.

## SDF in patients with Special needs and medically compromised adult populations:

Silver Diamine fluoride applications extend beyond treatment options to underserved populations or complex behavioral patients. SDF application is a minimally invasive approach that addresses critical needs in patients with special needs, patients with ongoing chemo or radiotherapy and even older adults with disparities that make access to complex dental treatments difficult. It is easy and safe to use, making it a great tool to bridge the gap to access proper dental care [22, 23].

## Children with special needs:

According to the American Dental Association, dental caries is the most frequent chronic disease in children worldwide. Feeding habits, biological, environmental and socioeconomic factors are involved in the development of early childhood caries; however, in patients with special needs, clinicians encounter an amalgamation of all those factors, but the most important one is their physical or intellectual despair, combined with lack of access to specialized care or the economic challenges of providing sedated treatments. Untreated oral conditions, such as dental caries, may lead to exacerbated systemic conditions [24]. SDF is well tolerated in special needs patients because, in the majority of those patients, we tend to observe anxiety, behavioral and physical difficulties that end up leaving the patient at high risk of untreated oral conditions. Approved in 2014, SDF has become an excellent alternative for caries prevention and arrest. It was primarily approved for dentinal sensitivity, but research indicates that SDF raises biofilm pH levels, contributing to reduced enamel demineralization [24]. SDF is a valuable noninvasive treatment for dental caries. It works effectively to arrest caries progression at a lower cost. It rapidly becomes a relief for caregivers, but most importantly, it reduces the risk of creating behavioral traumas for the children. A randomized control trial demonstrated that SDF had caries arrest of 94.5% in comparison with the combination of glass ionomer cement and varnish, which had a 90.1% arrest rate [25]. Studies have also shown that SDF can remineralize tooth structures within 6 weeks of its application. This suggests that SDF is valuable for stopping the progression of dental caries and promoting the healing of early lesions [26].

## SDF in compromised adults:

In medically compromised or functionally impaired patients who cannot tolerate conventional procedures, SDF, is the best possible alternative to an invasive surgical approach for arresting caries. Compromised adults include medically fragile patients with medical conditions such as chemo-radiotherapy for cancer, bisphosphonate usage, medication-related xerostomia, uncontrolled diabetes, and cardiovascular disease, patients with physical and intellectual disabilities, chronic conditions such as Addison's disease, lupus in the final stages and patients with limited access to dental care [27, 28]. Addressing oral health issues in older adults is a public health concern and SDF enhances the quality of life for this vulnerable population. Patients undergoing radiation therapy for head and neck often experience xerostomia and salivary gland dysfunction significantly increases the risk of radiation-induced caries. The annual application of SDF in these patients reduces the incidence of root caries lesions by up to 71% [29]. It is also recommended in caries arrest on non-restorable teeth, even more so if the patient is in hospice care. In that case, SDF can control the infection and ease some discomfort. SDF serves well in patients who cannot tolerate conventional treatment, who present dementia and behavioral or cognitive impairment, making it challenging to stay calm in the dental chair [27, 30].

## Emerging trends and long-term implications of silver diamine fluoride (SDF) Use in adult oral health care:

Silver diamine fluoride (SDF) has become a simple, painless, non-invasive and cost-effective treatment in recent years. For this reason, it is considered a desirable approach for preventing and managing dental caries in older adults, particularly those with reduced mobility, limited self-care capacity, or systemic conditions impairing healing. Scientific evidence has shown that an annual application of 38% SDF can significantly reduce the incidence of new lesions and arrest the progression of existing caries. This preventive strategy offers a predictable way to manage decay in adults and due to its minimal resource requirements, it is a highly accessible option for underserved populations, contributing to the growing trend of its use as a reliable alternative that benefits both community health and individual oral well-being [31]. As people age, they face several factors that increase their susceptibility to dental caries and gingival recession, raising the risk of root caries. From a public health perspective, the simplicity and accessibility of SDF present new opportunities to expand care delivery. Training caregivers, nurses and community health workers to identify carious lesions and assist with supervised SDF application could significantly improve oral health outcomes in institutionalized or remote populations. Mobile clinics, nursing homes and palliative care centers have increasingly adopted this model to help reduce disease burden and prevent emergency dental visits [32]. Adult patients often face economic, physical (e.g., mobility), or cognitive barriers to accessing routine dental services, especially when oral hygiene is limited or absent. The study by Mungur *et al.* (2023) evaluated different SDF application protocols in permanent teeth, focusing on older adults and comparing outcomes with other remineralizing agents. The results showed that even a single annual application of SDF produced outcomes comparable or superior to those of fluoride varnish and chlorhexidine [26]. SDF has proven to be well-tolerated, with strong clinical acceptance, economic feasibility and practical integration across public health initiatives and private practice. In light of this clinical reality, SDF emerges as an effective solution to reduce the need for invasive treatments, improve quality of life and preserve natural dentition in aging populations [33]. Beyond caries arrest, SDF strengthens residual tooth structure, reduces dentin hypersensitivity and lowers bacterial load by forming a protective barrier. Despite the characteristic black staining left on treated lesions, patient tolerance remains high, particularly in posterior teeth where aesthetics are not a primary concern. Only a minority reports discomfort or aesthetic dissatisfaction. This wide acceptance is attributed to the procedure's ease of use and non-invasive nature, making it especially beneficial for individuals with physical limitations or restricted access to conventional restorative care. This growing trend in using SDF offers a vital opportunity to address oral health disparities in communities facing structural challenges, such as the aging population. It also highlights the need for actionable strategies, such as expanding the scope of care beyond traditional dental offices by training auxiliary healthcare personnel to identify carious lesions and assisting in SDF's supervised application in broader care settings [34].

## SDF compared to other treatments:

Table 1 (see PDF) illustrates the comparison of SDF with other treatments. The analyses were carried out comparing the effectiveness of conventional restorative materials and other non-invasive agents like fluoride varnish or glass ionomer cements. Studies show that SDF offers superior antimicrobial action, longer-lasting caries arrest and greater ease of use in low-resource settings. While fluoride varnishes require frequent applications and GICs need mechanical preparation, SDF is applied with minimal intervention and has demonstrated arrest rates up to 80% with annual use (Table 1 - see PDF) [35, 36, 37, 38].

## Conclusion:

SDF is one of the most promising alternatives for adult dental care, especially in settings where prevention, simplicity and cost-effectiveness are critical. Its comparative advantages support the need for clear clinical guidelines to guide its rational use, with a strong focus on improving patient quality of life and ensuring the sustainability of the oral health system.

## References

[1] Yan IG (2022). Int Dent J..

[2] Chu CH (2012). Int J Paediatr Dent..

[3] Mei ML (2012). Dent Mater..

[4] Kettelkamp K (2025). Spec Care Dentist..

[5] Yee R (2009). J Dent Res..

[6] Horst JA (2016). J Calif Dent Assoc..

[7] Subbiah G (2018). J Int Soc Prevent Community Dent..

[8] Serna-Muñoz C (2025). Dent J (Basel)..

[9] Cleary J (2022). JDR Clin Trans Res..

[10] Mohapatra S (2025). J Healt Allied Sci..

[11] Oliveira BH (2018). J Dent Res..

[12] Zaffarano L (2022). Int. J. Environ. Res. Public Health.

[13] Osama S (2024). BMC Oral Health..

[14] Grandjean ML (2021). Swiss Dent J..

[15] Tan H (2010). J Dent Res..

[16] Noureldin A (2024). J Dent..

[17] Haiat A (2021). PLoS One..

[18] Shabbir J (2022). Biomed Res Int..

[19] Dioguardi M (2018). Eur J Dent.

[20] Zaeneldin A (2022). J Dent..

[21] Mathew MS (2025). Br Dent J..

[22] Crystal YO (2017). J Dent Res. Pediatr Dent..

[23] Brar A (2024). J Pharm Bioallied Sci..

[24] Contreras V (2017). Gen Dent..

[25] Ruff RR (2022). PLoS One..

[26] Hendre AD (2017). Gerodontology..

[27] Thomson WM (2004). Br Dent J..

[28] Magalhães AC (2017). Monogr Oral Sci..

[29] McReynolds D (2018). Evid Based Dent..

[30] Weyant RJ (2013). J Am Dent Assoc..

[31] Oliveira BH (2018). J Am Dent Assoc..

[32] Mungur A (2023). Clin Exp Dent Res..

[33] Zheng FM (2022). Dent. Sci Review..

[34] Castelo R (2021). Br Dent J..

[35] Seifo N (2019). BMC Oral Health..

[36] Baba MG (2021). Aust Dent J.

[37] De Moor RJG (2011). Comparative Study Clin Oral Investig..

[38] Griffin SO (2007). Am J Public Health..

